# Amphotericin B suppresses M2 phenotypes and B7-H1 expression in macrophages to prevent Raji cell proliferation

**DOI:** 10.1186/s12885-018-4266-0

**Published:** 2018-04-26

**Authors:** Jing Zhang, Dongqing Cao, Shuangquan Yu, Lingchao Chen, Daolin Wei, Chang Shen, Lin Zhuang, Qian Wang, Xiaoping Xu, Yin Tong

**Affiliations:** 1 Department of Hematology, The First People’s Hospital, Shanghai Jiaotong University, Shanghai, China; 2 Neurosurgical Immunology Laboratory, Department of Neurosurgery, Huashan Hospital, Fudan University, Shanghai, China; 3 Department of Hematology, Huashan Hospital, Fudan University, Shanghai, China

**Keywords:** Macrophage, Burkitt’s lymphoma, Amphotericin B, B7-H1

## Abstract

**Background:**

Macrophages in the tumor microenvironment play a critical role in tumorigenesis and anti-cancer drug resistance. Burkitt’s lymphoma (BL) is a B-cell non-Hodgkin’s lymphoma with dense macrophage infiltration. However, the role for macrophages in BL remains largely unknown.

**Methods:**

B7-H1, a transmembrane glycoprotein in the B7 family, suppresses T cell activation and proliferation and induces the apoptosis of activated T cells. The expression of B7-H1 in BL clinical tissues was determined by streptavidin-peroxidase immunohistochemistry. The mutual regulation between macrophages and BL Raji cells was investigated in a co-culture system. The cell proliferation and cell cycle distribution of Raji cells were determined using BrdU staining coupled with flow cytometry. CD163, CD204 and B7-H1 expression was assessed by flow cytometry and Western blot. Cell invasion was analyzed by Transwell assay. The expression of cytokines was detected by quantitative RT-PCR. Immunofluorescence and allogeneic T-cell proliferation assays were used to compare the expression of B7-H1, p-STAT6, or p-STAT3 and CD3+ T cell proliferation treated with or without amphotericin B.

**Results:**

B7-H1 was highly expressed in tumor infiltration macrophages in most clinical BL tissues. In vitro, Raji cells synthesized IL-4, IL-6, IL-10 and IL-13 to induce CD163, CD204 and B7-H1 expression in co-cultured macrophages, which in turn promoted Raji cell proliferation and invasion. Interestingly, antifungal agent amphotericin B not only inhibited STAT6 phosphorylation to suppress the M2 polarization of macrophages, but also promoted CD3+ T cell proliferation by regulating B7-H1 protein expression in macrophages.

**Conclusion:**

Amphotericin B might represent a novel immunotherapeutic approach to treat patients with BL.

**Electronic supplementary material:**

The online version of this article (10.1186/s12885-018-4266-0) contains supplementary material, which is available to authorized users.

## Background

B7-H1, also known as programmed death ligand − 1 (PD-L1) or CD274, is a member of the B7 family of immune regulatory molecules [1–3]. B7-H1 induces the apoptosis of tumor-specific T cells and T-cell unresponsiveness, contributing to T-cell anergy and exhaustion. Clinical studies have shown that the overexpression of B7-H1 on tumor cells and tumor-associated macrophages (TAMs) is related to poor prognosis in a variety of solid tumors including hepatocellular carcinoma [4]. However, the impact and regulatory mechanism of B7-H1 positive macrophages on the progression of B-cell lymphoma remains unclear.

Macrophages in the tumor microenvironment play a pivotal role in tumorigenesis and anti-cancer drug resistance [5–8]. Pro-inflammatory M1 macrophages are involved in antitumor immunity, while M2 macrophages provide a favorable microenvironment for tumor growth and angiogenesis [9–16]. Burkitt’s lymphoma (BL) is a B-cell non-Hodgkin’s lymphoma (NHL) characterized by the “starry sky” pattern due to dense macrophage infiltration, suggesting the important role for macrophages in the progression of BL. Conditioned medium from BL Raji cells was found to increase the expression of M2 macrophage CD163 and CD204 phenotypes in immature monocyte derived macrophages [17, 18]. However, the mechanism for the simulation of CD163 and CD204 expression remains unclear.

Co-culture experiments with immature monocyte derived macrophages and Raji cells were previously conducted using granulocyte macrophage-colony stimulating factor (GM-CSF) to induce immature macrophages [17–19]. It was found that immature macrophages in the setting of the tumor microenvironment provides a favorable microenvironment for tumor progression, supporting the findings that macrophages are a key component of the tumor microenvironment and play an important role in tumor progression [5, 20, 21]. Therefore, targeting macrophages in the tumor microenvironment is a promising strategy for the treatment of B-cell lymphoma.

Therapeutic approaches that targeting macrophages in tumor tissues have been reported to greatly improve the outcome of gliomas, prostate cancer and breast cancer [22, 23]. Amphotericin B is an activator of monocytoid cells. Sarkar [24] found that human microglia treated with amphotericin B suppressed brain tumor initiating cells. In addition, amphotericin B induced rat primary microglia to express iNOS protein and increased the mRNA expression of pro-inflammatory cytokines IL-1β and TNF-α [25]. However, whether amphotericin B suppresses the growth and proliferation of BL cells through inhibiting macrophages in the tumor microenvironment remains to be determined.

In the present study, using a previously established co-culture system, we evaluated the effect of amphotericin B on human immature macrophages. It was found that amphotericin B restrained immature macrophages from stimulating the growth, invasiveness and immune escape of Raji cells. Furthermore, it was revealed that amphotericin B inhibited STAT6 phosphorylation and promoted CD3+ T-cell proliferation.

## Methods

### Patients and immunohistochemistry (IHC) for B7-H1

A total of seven patients diagnosed with BL at the First Affiliated Hospital of Zhejiang University were enrolled in this study. Written informed consent for experimental use of specimens was obtained from all patients. The protocols in the present study received approval from the ethics committee of the Institutional Review Board of Huashan Hospital of Fudan University (Permit Number: 2013–034). The BL clinical specimens were prepared as paraffin-embedded sections. The expression of B7-H1 in BL clinical tissues was determined using a streptavidin-peroxidase immunohistochemistry kit (Cell Signaling Technology). Five random fields were observed for each specimen under high magnification (400×) to count the number of positive cells. Staining intensity was scored as follows: 0 (no staining), 1+ (weak or equivocal staining), 2+ (moderate staining), or 3+ (strong staining). Positive B7-H1 staining was defined per specimen by a 5% expression threshold of total TAMs.

### Double immunofluorescence staining for CD68 and B7-H1 in BL tissues

The sections of BL clinical specimens were deparaffinized, rehydrated and then incubated in 0.3% H2O2 in TBS for 20 min to suppress endogenous peroxidase activity. The antigen retrieval buffer were preheat to 95 °C, then heated in a staining jar which is placed in a water bath at 95 °C for 20 min. The vessel removed and cooled naturally. The slides were washed and blocked in 10% normal serum with 1% BSA in TBST for 30 min at room temperature. The slides were drained and incubated with primary antibodies (CD68 1:400, B7-H1 1:50) (Abcam) overnight at 4 °C. The sections were washed in TBST at three times for 5 min and incubated with secondary antibody in 1% BSA in TBST for 1 h at room temperature in the dark. The secondary antibody solution were decanted and washed three times in TBST. Then the slides were incubated on 1 μg/mL DAPI for 5 min. After washed the slides three times in TBST, we sealed sheet with anti-quenching sealing liquid. Photographs were taken with a microscope.

### Cells and reagents

BL cell line Raji cells were purchased from the American Type Culture Collection, and cultured in RPMI-1640 medium (Gibco) supplemented with 10% fetal bovine serum (FBS, Gibico) in an incubator at 37 °C with 5% CO_2_. Normal human peripheral blood mononuclear cells (PBMCs) were isolated from healthy volunteer donors, from whom a written informed consent for experimental use of specimens was obtained. Immature macrophages were induced, as previously reported [17, 18]. CD14+ monocytes were isolated from normal human PBMCs using CD14-microbeads (Miltenyi Biotec). The sorting purity of CD14+ monocytes was higher than 95%. Monocytes were cultured with RPMI-1640 medium supplemented with 10% FBS and stimulated with 5 ng/ml of GM-CSF (R&D) for five days. CD11b, CD11c, CD68, CD1a and CD83 were used for the identification of immature macrophages from dendritic cells (DCs) by flow cytometry.

Amphotericin B was purchased from Sigma and dissolved in DMSO. In order to reduce the impact of DMSO on immature macrophages, the concentration of DMSO in the cell culture was lower than 0.2%. CCK-8 assay was used to assess the effects of amphotericin B on the cell viability of immature macrophages.

### Co-culture experiments

Co-culture experiments were performed, as previously reported [17, 18]. Raji cells (5 × 10^4^ cells/well) and immature macrophages (6 × 10^4^ cells/well) were co-cultured in DMEM medium (Gibco) supplemented with 2% FBS for 72 h in 0.4-μm Transwell chamber dishes (Corning). For amphotericin B treatment, immature macrophages were first treated with 1 μM of amphotericin B for 72 h, and subjected to co-culture experiments without amphotericin B. In order to rule out the effect of amphotericin B directly on Raji cells, cells washed once after drug treatment.

### Proliferation assays and cell cycle analysis

The cell proliferation and cell cycle distribution of Raji cells were determined using BrdU Flow Kits (BD Pharmingen), according to manufacturer’s instructions. Then, 10 μL of BrdU solution (1 mM) was directly added to the tissue culture medium and incubated for 45 min. Next, cells were fixed and permeabilized for 30 min at room temperature, washed twice with ice-cold phosphate-buffered saline (PBS) and treated with DNase. After washing with PBS for three times, 100 μl of staining buffer containing 7-AAD was added to each well. Cells were analyzed on a FACScan flow cytometer (BD Biosciences), and data were interpreted using the Flowjo software (Tree Star). Each experiment was performed in triplicate.

### Flow cytometry

CD14, CD68, CD11b, CD11c, CD83 and CD1a fluorochrome-conjugated antibodies (BD Biosciences) were used to stain the immature macrophages in the dark at 4 °C for 15 min. After co-culture experiments for 72 h, immature macrophages were obtained by incubating with CD163 (Miltenyi Biotec), CD204 (Miltenyi Biotec), CD206 (Miltenyi Biotec) and B7-H1 (eBioscience) fluorochrome-conjugated antibodies in the dark at 4 °C for 15 min, followed by incubation with iNOS antibody (Abcam) and fluorescently-labeled secondary antibody. Cells were analyzed on a FACScan flow cytometer (BD Biosciences), and data were interpreted using the Flowjo software (Tree Star). Each experiment was performed in triplicate.

### Western blot analysis

Immature macrophages following the co-culture experiments were washed with PBS and lysed with RIPA (Beyotime) in the presence of 1 mM of PMSF (Beyotime). Protein samples were separated by 10% SDS-PAGE and transferred onto PVDF membranes (Immobilon-P membrane; Millipore). After incubation in 5% fat-free milk at room temperature for two hours to block non-specific binding, the membranes were incubated with primary antibodies (1:1000) in TBS-Tween (TBST, 0.05% Tween-20 in TBS) at 4 °C overnight. iNOS, CD163, CD204, CD206, p-STAT3 and STAT3 antibodies were purchased from Abcam. B7-H1, p-STAT6, STAT6 and β-Actin antibodies were purchased from Cell Signaling Technology. Next, these membranes were washed three times with TBST and incubated with the secondary antibody (1:5000) for one hour at room temperature. The membranes were washed three times with TBST and visualized by enhanced chemiluminescence using a SuperSignal West Pico Trial Kit (Thermo Scientific, Massachusetts, USA).

### Tumor invasion assay

Transwell chamber dishes (8 μm, Corning) were used to perform the tumor invasion assay. Raji cells (1 × 10^5^ cells/well) were placed in the upper chamber, while immature macrophages (6 × 10^4^ cells/well) were placed in the lower chamber. After incubation for 48 h, non-migratory cells on the upper surface of the insert were gently removed using a cotton-tipped swab, and cells that entered the lower surface of the membrane were fixed and stained with crystal violet at room temperature for 20 min. Light microscopy was used to count cells that invaded through the Matrigel coating. For quantification, five randomly selected fields on the lower side of the insert were photographed. Each experiment was performed in triplicate.

### Quantitative real-time PCR (qRT-PCR)

Total RNA was extracted using Trizol Reagent (Invitrogen), according to manufacturer’s instructions. Reverse transcription was performed using PrimeScript RT Master Mix (TaKaRa), and qRT-PCR was performed using SYBR Premix Ex Taq (TaKaRa) on a 7500 Real-Time PCR System (ABI, Grand Island, NY, USA). All reactions were performed in triplicate. The primers used for RT-PCR analysis: IL-4 Forward: 5’-GACATCTTTGCTGCCTC-3′, Reverse: 5’-TACTCTGGTTGGCTTCCTTCA-3′; IL-6 Forward: 5’-AAAGAGGCACTGGCAGAAAA-3′, Reverse: 5’-TTTCACCAGGCAAGTCTCCT-3′; IL-10 Forward: 5’-ACGGCGCTGTCATCGATT-3′, Reverse: 5’-GGCATTCTTCACCTGCTCCA-3′; IL-13 Forward: 5’-ATCCTCTCCTGTTGGCAC-3′, Reverse: 5’-CTGGTTCTGGGTGATGTTGAC-3′; IL-1β Forward: 5’-CGACACATGGGATAACGA-3′, Reverse: 5’-CGCAGGACAGGTACAGATTC-3′; IL-12 Forward: 5’-TTGCCTAAATTCCAGAGAGA-3′, Reverse: 5’-AGCTTTGCATTCATGGTCTTG-3′; TNF-α Forward: 5’-AGCCCATGTTGTAGCAAACC-3′, Reverse: 5’-TGAGGTACAGGCCCTCTGAT-3′; GAPDH Forward: 5’-TGCTGAGTATGTCGTGGAGTCT-3′, Reverse: 5’-AATGGGAGTTGCTGTTGAAGTC-3′. Each experiment was performed in triplicate.

### Phagocytosis assay

The phagocytic function of immature macrophages was evaluated by exposing cells to pHrodo *E. coli* BioParticles (Life technologies) for at least 15 min at 37 °C, according to manufacturer’s instructions. The percentage of macrophages that phagocytosed pHrodo *E. coli* BioParticles conjugates was analyzed using a flow cytometer equipped with a 488-nm argon-ion laser. Each experiment was performed in triplicate.

### Immunofluorescence assay

The cell suspension of immature macrophages was dropped on poly-lysine-treated glass slides, fixed with 0.2% Triton-100 for 20 min, and blocked by 3% BSA for one hour. B7-H1, p-STAT6, or p-STAT3 antibody (1:100) was added and incubated at 4 °C overnight. Cells were washed with TBST and added with the secondary antibody (1:1000). After one hour, DAPI was added to stain the cell nuclei. Photographs were taken with a microscope.

### Allogeneic T-cell proliferation assay

CD3+ and CD4+ T cells were enriched from PBMCs obtained from healthy donors using CD3-microbeads (Miltenyi Biotec) and CD4-microbeads (Miltenyi Biotec), respectively, according to manufacturer’s protocol. CD3+ T cells were labeled with CFSE and washed three times with complete medium. Enriched T cells (6 × 10^5^ cells/well) were cultured in RMPI-1640 medium supplemented with 10% FBS at a ratio of 10:1 with immature macrophages after co-culture experiments in 24-well plates with or without 10 mg/ml of anti-B7-H1 (clone MIH1) or mouse IgG1 isotype control mAbs (eBioscience). After 72 h, CD3+ T cells were harvested for cell proliferation assay using BrdU Flow Kits. CD4+ T cells were harvested to detect the percentage of CD4 + CD25 + Foxp3+ T cells using a Human Regulatory T cell Staining Kit (eBioscience), according to manufacturer’s instructions. All T cells were analyzed on a FACScan flow cytometer (BD Biosciences), and data were interpreted by the Flowjo software (Tree Star). Each experiment was performed in triplicate.

### Statistical analysis

Results were presented as mean ± standard deviation (SD). Paired *t*-test was used for two-group comparisons and one-way ANOVA was performed to compare the means of three or more variables. All statistical analyses were performed using Stata 9.0. *P* < 0.05 was considered statistically significant.

## Results

### B7-H1 is expressed in TAMs of BL tissues

**Fig. 1:**
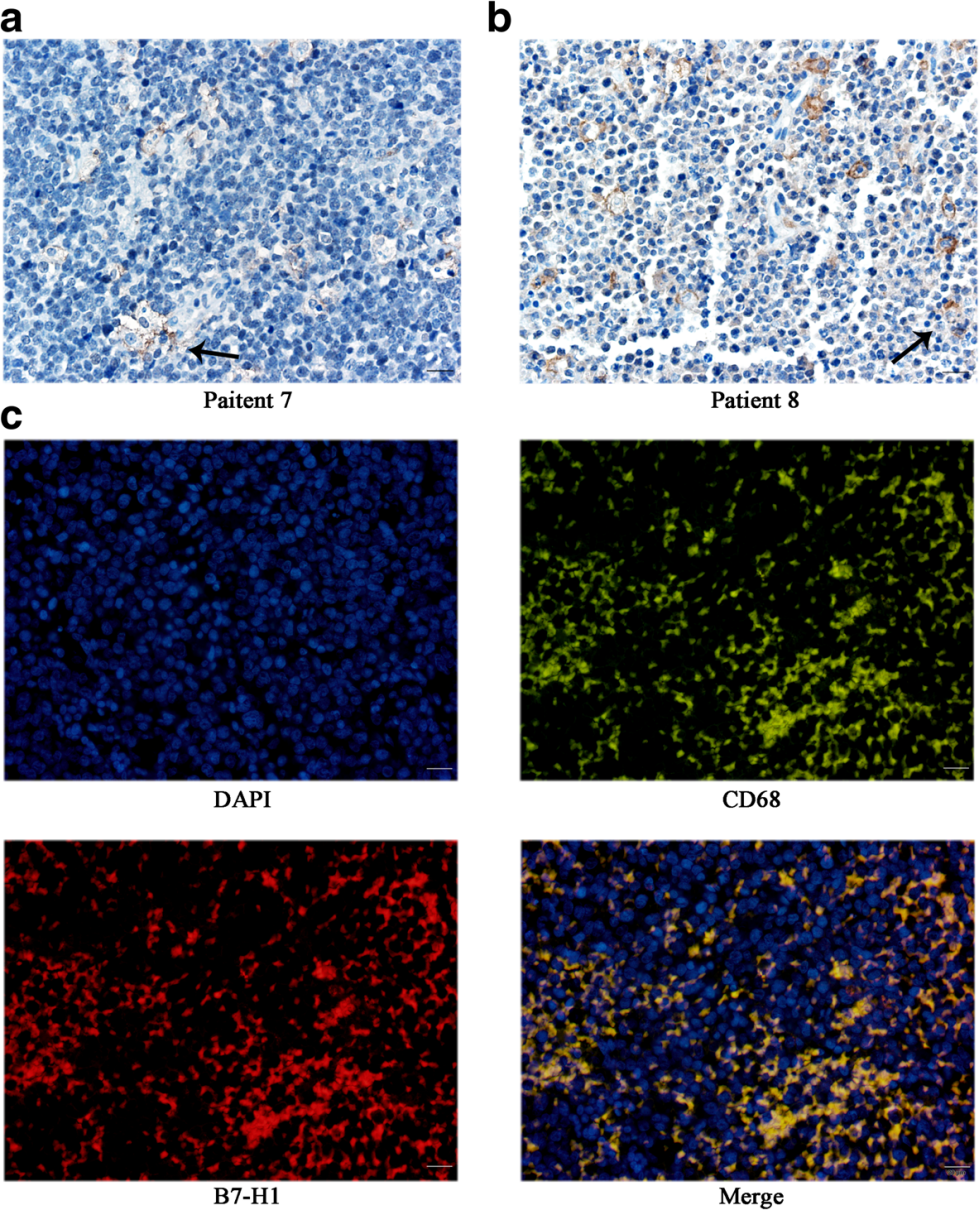
B7-H1 was expressed in tumor-associated macrophages (TAMs) of BL tissues. **a** and **b** Immunohistochemical analysis of B7-H1+ cells in tumor tissues (400×). B7-H1 positive TAMs that ingested apoptotic tumor cells are indicated by arrows. Scale 20 μm. **c** Double immunofluorescence staining showed a high degree of overlap expression with B7-H1 and CD68. Scale 20 μm

The clinicopathological features of patients with BL are summarized in Table 1. The median age of patients was 41 years (range: 26–68 years). All patients were diagnosed at advanced stages (III and IV). B7-H1 expression was assessed in BL clinical tissues obtained from patients by IHC (Fig. 1a and b). It was found that 71.43% (5/7) of BL tissues were positive for B7-H1 staining in the cytoplasm and membrane of TAMs, in which the B7-H1 staining intensity score was 3+ in one case, 2+ in three cases and 1+ in one case. However, there was no positive B7-H1 staining in tumor cells. Double immunofluorescence staining was performed to locate B7-H1 expression in BL tissues. As shown in Fig. 1c, we found CD68 macrophages strongly expressed B7-H1 protein and tumor cells were negative.

**Table 1:** The clinicopathological features of patients with BL

ID	Histopathology sources	Gender	Age-ranges (years)	Stage	B7-H1+ (number/ high magnification)
1	Greater omentum	Female	18–60	IV	0
2	Lymph node	Male	18–60	IV	14
3	Lymph node	Male	18–60	IV	0
4	Ovary and oviduct	Female	18–60	IV	19
5	Pancreas	Male	18–60	IV	7
6	Lymph node	Male	18–60	IV	8
7	Lymph node	Female	> 60	III	24

### Macrophages promote the proliferation of Raji cells while Raji cells enhance B7-H1 expression in macrophages

**Fig. 2:**
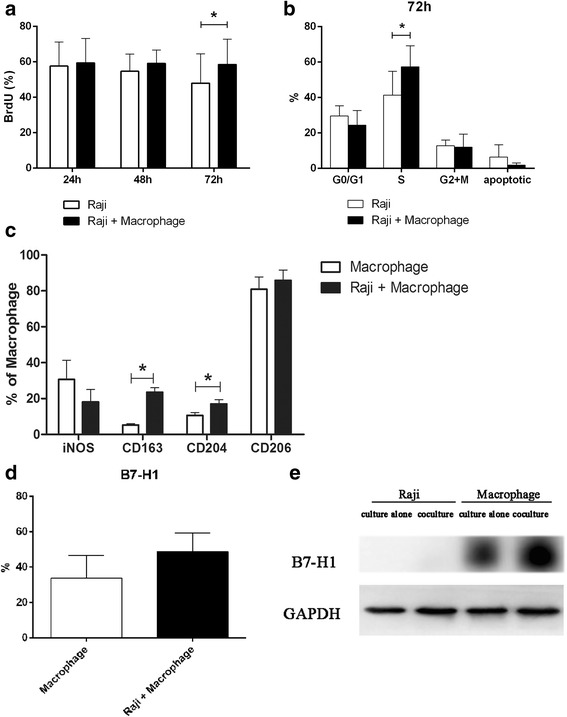
Macrophages promote the proliferation of Raji cells while Raji cells enhance B7-H1 expression in macrophages (**a**) Raji cells were cultured with or without macrophages for 24, 48 and 72 h, followed by the analysis of the cell proliferation of Raji cells by BrdU incorporation assay (*P < 0.05). **b** Raji cells were cultured with or without macrophages for 72 h, and the cell cycle distribution of Raji cells was analyzed by flow cytometry (*P < 0.05). **c** Macrophages were cultured with or without Raji cells for 72 h, followed by the analysis of iNOS, CD206, CD163 and CD204 by flow cytometry. M2 phenotypes (CD163 and CD204) increased in macrophages (*P < 0.05). **d** Macrophages were treated as c, and B7-H1 expression was assessed by flow cytometry (*P < 0.05). **e** Macrophages were treated as c, and B7-H1 expression was assessed by western blot

In order to explore whether macrophages play a role in BL, Raji cells were co-cultured with immature monocyte derived macrophages. That is, CD14+ monocytes were isolated from PBMCs using CD14-microbeads, and cultured with 5 ng/mL of GM-CSF for five days. Cells were analyzed for CD14, CD11b, CD11c, CD1a, CD83 and CD68 by flow cytometry. Most of the macrophages were positive for CD11b and CD11c, with a low expression of CD68 (Additional file 1: Figure S1). GM-CSF also induced monocytes to DCs. CD83 is a mature phenotype related to the degree of maturation of DCs, and CD1a is a DC-specific surface marker that reflects the number of DCs. The low expression of CD83, CD1a and CD68 revealed that primary human monocytes differentiated to macrophages (Additional file 1: Figure S1). Next, Raji cells were cultured with or without macrophages for 24, 48 and 72 h, followed by the proliferation analysis of Raji cells by BrdU incorporation assay. Results revealed that macrophages promoted the percentage of BrdU positive Raji cells after 72 h (Fig. 2a, *P*< 0.05). Moreover, cells in the S phase were significantly higher in Raji cells co-cultured with macrophages than cells that were not co-cultured with macrophages (Fig. 2b, *P*< 0.05).

M2 macrophage infiltration has been reported to promote tumor progression in a variety of tumors [17, 26, 27]. In order to test whether Raji cells in turn affects the differentiation of macrophages, macrophages were cultured with or without Raji cells for 72 h; followed by the analysis of iNOS, CD206, CD163 and CD204. The expression of M2 phenotypes CD163 and CD204 significantly increased in macrophages co-cultured with Raji cells for 72 h (Fig. 2c, *P*< 0.05). Next, B7-H1 protein expression was analyzed in macrophages cultured with or without Raji cells using flow cytometry, and it was found that the expression of B7-H1 was significantly higher in macrophages co-cultured with Raji cells for 72 h than macrophages cultured alone (Fig. 2d, *P*< 0.05). Further western blot analysis revealed that there was no B7-H1 expression in Raji cells, and co-culturing with Raji cells enhanced the expression of B7-H1 in macrophages (Fig. 2e).

### IL-4 and IL-13 increases the phosphorylation of STAT6 and the expression of B7-H1 in macrophages

**Fig. 3:**
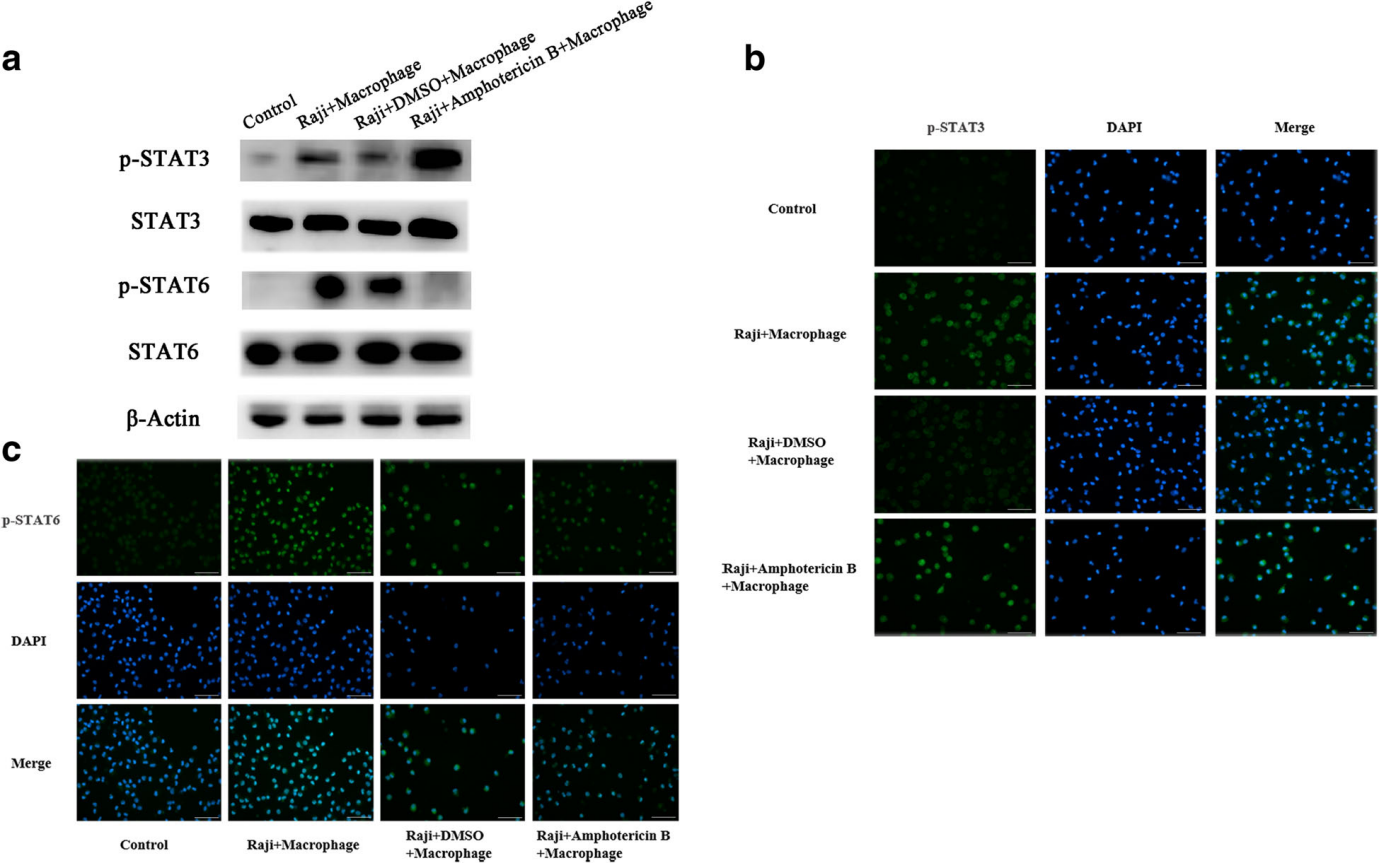
Amphotericin B abolished Raji cells induced STAT6 phosphorylation in macrophages. **a** Raji cells were cultured with or without macrophages in the presence or absence of amphotericin B (1 μM) for 72 h, and STAT3 and STAT6 phosphorylation in macrophages were assessed by western blotting. Amphotericin B only inhibited the expression level of p-STAT6. **b** Cells were treated as a, and STAT3 phosphorylation in macrophages was detected by immunofluorescence (400×). Scale 50 μm. **c** Cells were treated as a, and STAT6 phosphorylation in macrophages was detected by immunofluorescence (400×). Scale 50 μm. Control: macrophages alone without Amphotericin B treatment; Raji + Macrophages: macrophages without Amphotericin B treatment co-cultured with Raji cells; Raji + DMSO + Macrophages: macrophages with DMSO treatment first and then co-cultured with Raji cells; Raji + Amphotericin B + Macrophages: macrophages with Amphotericin B treatment first and then co-cultured with Raji cells

**Fig. 4:**
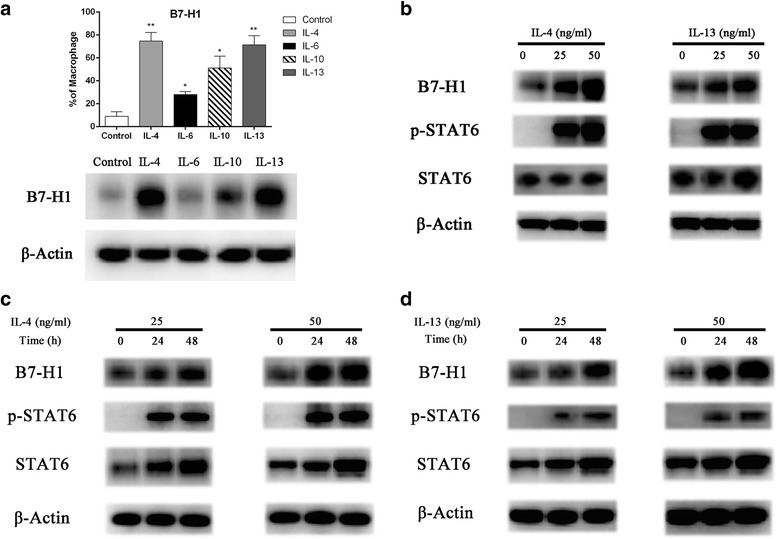
IL-4 and IL-13 increased the expression of B7-H1 in macrophages. **a** Macrophages were treated with IL-4, IL6, IL10 and IL-13 for 24 h. The analysis of B7-H1 levels was determined by flow cytometry (upper panel) and western blotting (lower panel), respectively. **b** Macrophages were treated with 25 or 50 ng/ml of IL-4 or IL-13 for 24 h. p-STAT6 and B7-H1 were determined by immunoblotting. **c** Macrophages were treated with 25 or 50 ng/ml of IL-4 for 24 and 48 h, and p-STAT6 and B7-H1 were determined by immunoblotting. **d** Macrophages were treated with 25 or 50 ng/ml of IL-13 for 24 and 48 h, and p-STAT6 and B7-H1 were determined by immunoblotting. *P < 0.05, ** *P* < 0.01

B7-H1 expression in tumors and circulating monocytes was reported to be regulated by certain cytokines and STAT signaling pathways, including STAT6 and STAT3 [28–30]. In order to determine the mechanism by which Raji cells increase B7-H1 expression in macrophages, western blot and immunofluorescence was performed to assess STAT3 and STAT6 phosphorylation in macrophages after co-culture. Immunoblotting results revealed that Raji cells promoted STAT3 and STAT6 phosphorylation in macrophages (Fig. 3a). Similarly, immunofluorescence analyses demonstrated that the staining of pSTAT3 (Fig. 3b) and pSTAT6 (Fig. 3c) was higher in macrophages co-cultured with Raji cells, compared to macrophages cultured alone. STAT6 plays a central role in IL-4 and IL-13 mediated biological responses [31, 32]. Next, macrophages were treated with IL-4, IL-6, IL-10 and IL-13 for 24 h, and the expression B7-H1 was determined by RT-PCR and immunoblotting, respectively. Interestingly, it was found that all cytokines increased the mRNA levels of B7-H1 in macrophages with greater induction by IL-4 and IL-13 (Fig. 4a; *P* < 0.01 and *P* < 0.01, respectively). Consistently, western blot results revealed that all cytokines increased the protein levels of B7-H1 in macrophages with greater induction by IL-4 and IL-13 (Fig. 4b). Then, macrophages were treated with 25 or 50 ng/ml of IL-4 or IL-13 for 24 h, and the protein expression of p-STAT6 and B7-H1 was determined by immunoblotting. It was found that IL-4 and IL-13 increased p-STAT6 and B7-H1 in macrophages in a concentration dependent manner (Fig. 4b). Further treatment of macrophages with 25 or 50 ng/ml of IL-4 or IL-13 for 24 and 48 h revealed that IL-4 and IL-13 increased p-STAT6 and B7-H1 in macrophages in a time dependent manner (Fig. 4c and d).

These results indicate that IL-STAT signaling simulates B7-H1 expression in macrophages, which in turn promotes tumor progression. These findings suggest that targeting macrophages may be effective in suppressing lymphoma growth. Amphotericin B has been reported to induce rat primary microglia to express iNOS protein, a M1 phenotype. In order to test whether treating macrophages with amphotericin B inhibits the growth of lymphoma cells, macrophages were treated with increasing concentrations of amphotericin B for 24, 48 and 72 h, and cell viability was assessed. Low doses (1 μM) of amphotericin B are not toxic to macrophages (Additional file 2: Figure S2). Next, the co-cultured macrophages were treated with Raji cells in the presence or absence of amphotericin B (1 μM) for 72 h. It was found that amphotericin B inhibited the phosphorylation of STAT6, but not STAT3, in the co-cultured macrophages (Fig. 3a and c).

### Amphotericin B abolishes the macrophage-mediated stimulation of the proliferation and invasiveness of Raji cells

**Fig. 5:**
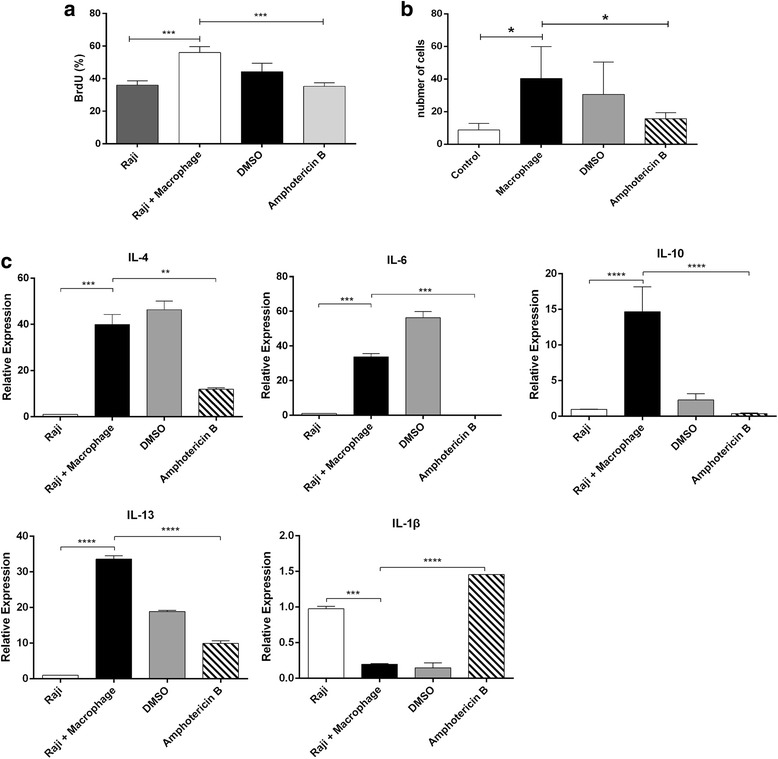
Amphotericin B inhibited the macrophage-mediated promotion of the proliferation and invasiveness of Raji cells. **a** Raji cells were co-cultured with macrophages pretreated with DMSO or amphotericin B, followed by the analysis of the cell proliferation of Raji cells by BrdU incorporation assay (***P < 0.001). **b** Cells were treated as a, and Raji cell invasion was assessed by Transwell assay (*P* < 0.05). **c** Cells were treated as a for 72 h, and the mRNA expression of IL-4, IL-6, IL-10, IL-1β and IL-13 in Raji cells was determined by RT-PCR. *P < 0.05, **P < 0.01, ***P < 0.001, ****P < 0.0001. Macrophage: macrophages alone without Amphotericin B treatment; Raji: Raji alone; Raji + Macrophages: macrophages without Amphotericin B treatment co-cultured with Raji cells; DMSO: macrophages with DMSO treatment first and then co-cultured with Raji cells; Amphotericin B: macrophages with Amphotericin B treatment first and then co-cultured with Raji cells

The suppression of p-STAT6 by amphotericin B in macrophages suggest that amphotericin B may prevent the promotion of Raji cell proliferation in co-cultures with macrophages. That is, Raji cells were co-cultured with macrophages pretreated with DMSO or amphotericin B. BrdU incorporation assay revealed that macrophages treated with amphotericin B failed to promote the proliferation of Raji cells (Fig. 5a, *P*< 0.05). Further Transwell assay revealed that amphotericin B counteracted the ability of macrophages to promote Raji cells to pass through the membrane pores coated with Matrigel (Fig. 5b, *P*< 0.05). RT-PCR analyses demonstrated that macrophages increased the mRNA levels of IL-1β. IL-4, IL-6, IL-10 and IL-13 (Fig. 5c, *P*< 0.001, *P* < 0.001, *P* < 0.0001 and *P* < 0.0001, respectively), and reduced the levels of IL-1β (Fig. 5c, *P*< 0.001) in Raji cells co-cultured for 72 h. More importantly, macrophages treated with amphotericin B reduced the mRNA levels of IL-4, IL-6, IL-10 and IL-13 (Fig. 5c, *P*< 0.01, *P* < 0.001, *P* < 0.0001 and *P* < 0.0001, respectively) and increased the mRNA expression of IL-1β in Raji cells (Fig. 5c, *P*< 0.0001).

### Amphotericin B suppresses M2 polarization and enhances the phagocytic function of macrophages

**Fig. 6:**
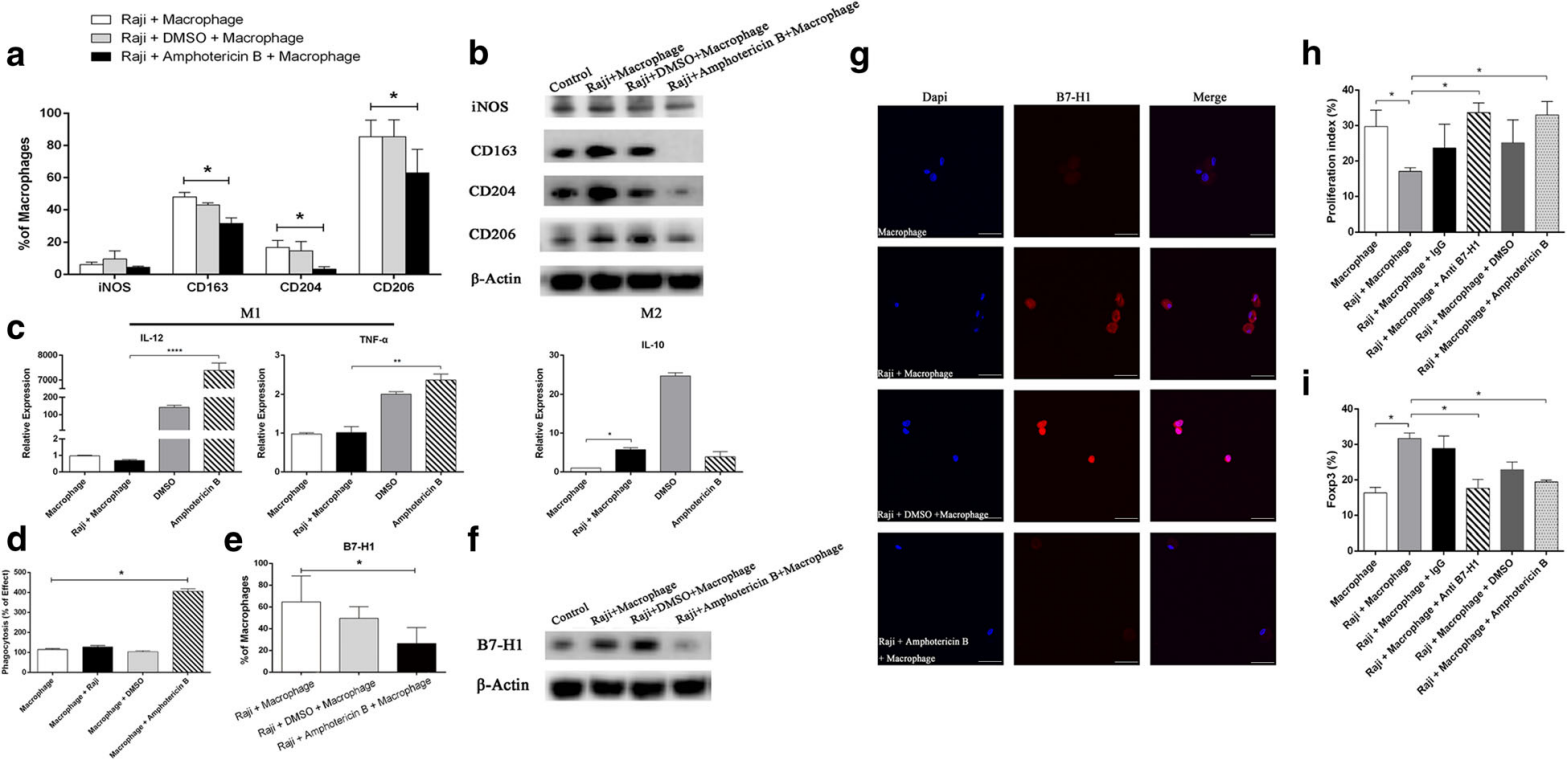
Amphotericin B reduced the expression of CD163, CD204, CD206 and B7-H1, and enhanced the phagocytic function of co-cultured macrophages. **a** Raji cells were co-cultured with macrophages treated with DMSO or amphotericin B for 72 h. iNOS, CD163, CD204 and CD206 protein expression in macrophages were determined by flow cytometry. Amphotericin B reduced the expression of M2-related phenotypes CD163, CD204 and CD206 on the surface of the co-cultured macrophages (*P* < 0.05). **b** Cells were treated as a, and the protein levels of iNOS, CD163, CD204 and CD206 in macrophages were determined by immunoblotting. **c** Cells were treated as a, and the mRNA expression of IL10, IL-12 and TNF-α in the co-cultured macrophages was detected by RT-PCR (*P* < 0.01). **d** Amphotericin B enhanced the phagocytic function of macrophages. **e** Amphotericin B reduced B7-H1 expression on the surface of co-cultured macrophages (*P* < 0.05). **f** Amphotericin B reduced B7-H1 protein expression in co-cultured macrophages. **g** Confocal microscopy revealed that amphotericin B reduced B7-H1 expression in co-cultured macrophages (600×). Scale 50 μm. **h** Raji cells were co-cultured with macrophages treated with DMSO or amphotericin B for 72 h. Then, these macrophages were co-cultured with CD3+ T cells in 24-well plates with or without 10 mg/ml of anti-B7-H1 (clone MIH1) or mouse IgG1 isotype control mAbs (eBioscience). After 72 h, CD3+ T cells were harvested for cell proliferation assay using a BrdU Flow Kit. Co-cultured immature macrophages significantly inhibited CD3+ T cell proliferation. Macrophages treated with amphotericin B restored CD3+ T cell proliferation (*P* < 0.05), which is consistent with the results of the anti-B7-H1 treatment. **i** Cells were treated as h, and CD4+ T cells were harvested for the detection of the percentage of CD4 + CD25 + Foxp3+ T cells by flow cytometry. Co-cultured macrophages significantly induced Foxp3 expression in Treg cells. Amphotericin B treatment and anti-B7-H1 antibody inhibited the expression of Foxp3 in Treg cells co-cultured with macrophages (*P* < 0.05), which was consistent with the results of the anti-B7-H1 treatment. *P < 0.05, **P < 0.01, ***P < 0.001, ****P < 0.0001

IL-4, IL-6, IL-10 and IL-13 have been reported to induce M2 macrophages, and the M1 phenotype is driven by IL-1β. These above results suggest that amphotericin B may reduce the ratio of M2 macrophages. To this, Raji cells were co-cultured with macrophages pretreated with DMSO or amphotericin B. Flow cytometry analysis revealed that amphotericin B significantly reduced the expression of M2-related phenotypes CD163, CD204 and CD206 (Fig. 6a, *P*< 0.05), which was confirmed by western blot analysis (Fig. 6b). However, amphotericin B did not increase iNOS expression in macrophages (Fig. 6a and b). M1 macrophages are characterized by the production of pro-inflammatory factors such as IL-12 and TNF-α. The results of the present study revealed that amphotericin B increased IL-12 and TNF-α mRNA expression in co-cultured macrophages (Fig. 6c; *P* < 0.0001 and *P* < 0.01, respectively). Furthermore, amphotericin B enhanced the phagocytic function of macrophages (Fig. 6d, *P*< 0.01). Taken together, these results suggest that amphotericin B may indirectly inhibit the proliferation and invasiveness of Raji cells by suppressing the M2 polarization of macrophages.

### Amphotericin B promotes CD3+ T cell proliferation and inhibits Foxp3 expression in CD4+ T cells by regulating the B7-H1 protein expression of macrophages

B7-H1 plays a role in the regulation of immune responses and peripheral tolerance. B7-H1 induces the apoptosis of tumor-specific T cells and T-cell unresponsiveness [1–3]. It was found that amphotericin B reduced B7-H1 expression in co-cultured immature macrophages (Fig. 6e, f and g; *P* < 0.05). Finally, CD3+ or CD4+ enriched T cells were cultured at a ratio of 10:1 with macrophages after co-culture experiments with or without 10 mg/mL of anti-B7-H1 or mouse IgG1 isotype control mAbs. After 72 h, CD3+ T cells were harvested for cell proliferation assay using a BrdU Flow Kit. CD4+ T cells were harvested to detect the percentage of CD4 + CD25 + Foxp3+ T cells using a Human Regulatory T cell Staining Kit. It was found that amphotericin B promoted CD3+ T cell proliferation and inhibited Foxp3 expression in CD4+ T cells by regulating the B7-H1 protein expression of immature macrophages (Fig. 6h and i, *P*< 0.05).

## Discussion

Although the tumor microenvironment exerts host immune surveillance during the earliest stages of tumor growth, immune escape remains as a thorny problem during tumor progression. TAMs have been reported to provide a favorable microenvironment for tumor growth, tumor survival, angiogenesis and immune escape in response to microenvironmental challenges [33–36]. The progression of hematopoietic and lymphoid cancers is associated with the activation of the B7-H1/PD-1 pathway, leading to immune disorders [37]. Blocking B7-H1/PD-1 can reduce the mortality of AML in mice [38]. BL is a B-cell NHL characterized by dense macrophage infiltration. However, the role for B7-H1 in a BL microenvironment remains to be determined. We found that B7-H1 is expressed in TAMs of BL specimens. In the present study, in order to observe whether the differentiation of macrophages associated with cytokines are secreted by tumor cells, immature monocyte derived cells were cultured with Raji cells instead of M2 macrophages induced by M-CSF. In the indirect co-culture system in vitro, macrophages promoted the proliferation of Raji cells. We further found that the co-culture system induced M2 polarization and the B7-H1 expression of macrophages, in order to provide a favorable microenvironment for tumor growth, and spread instead of exerting the anti-tumor effect of macrophages. Taken together, our results indicate that Raji cells not only induce the M2 polarization for tumor progression, but also promote B7-H1 expression in immature macrophages for tumor immune escape. These results suggest that macrophages may serve as an important ingredient in tumor immune escape, and targeting macrophages is a new therapeutic strategy to BL.

Amphotericin B has been reported to regulate the antitumor effect of macrophages by means of toll-like receptor signaling [25]. As a common antifungal agent, the role of amphotericin B in immune regulation remains unclear. The dose of amphotericin B (1 μM) used in our research was much lower than that presently used in clinic to treat patients with fungal infections. Moreover, amphotericin B revealed no signs of toxicity to immature macrophages in the present study. We observed that amphotericin B inhibited M2 polarization by suppressing STAT6 phosphorylation in immature macrophages. The STAT6 signaling pathway is activated by IL-4 and IL-13 to skew macrophage function towards the M2 phenotype [39]. CD163 is one of the most common phenotypes of M2 macrophages. A number of studies have reported that higher percentages of CD163+ tumor-infiltrating macrophages were significantly correlated with the poor clinical course of lymphoma [6, 7, 26, 40–42]. Our results revealed that amphotericin B inhibited the proliferation and invasiveness of Raji cells probably by means of depriving immature macrophages of M2 polarization in studies in vitro.

**Fig. 7:**
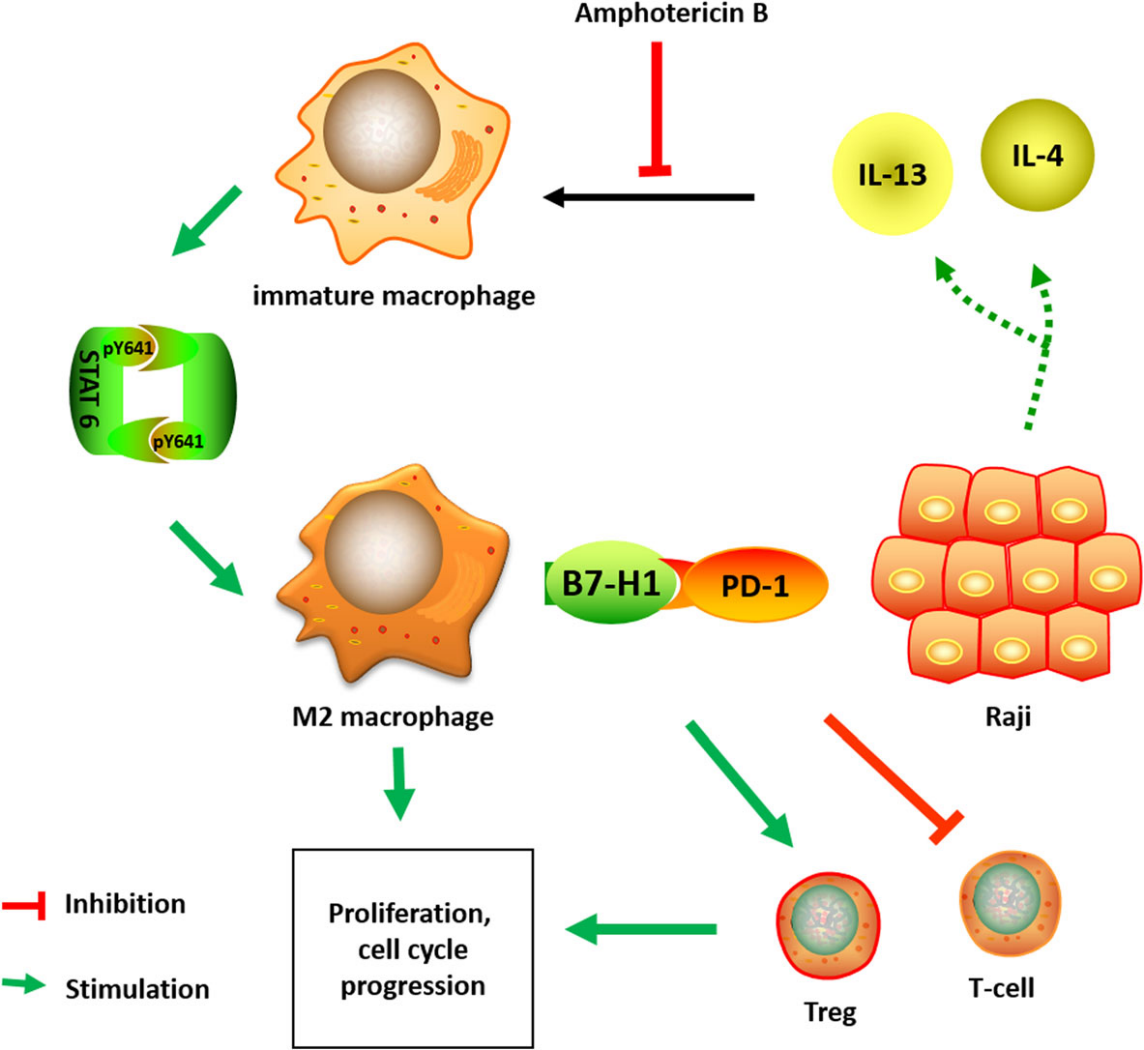
A diagram shows the crosstalk between Raji cells and macrophages. Picture material from Science Slides

Importantly, we revealed that amphotericin B also promoted CD3+ T cell proliferation and inhibited Foxp3 expression in CD4+ T cells by regulating the B7-H1 protein expression of immature macrophages. To the best of our knowledge, this is the first study that reported the B7-H1 regulation effect of amphotericin B. Our findings reveal a new insight into the prevention of tumor immune escape. We further investigated the mechanism of B7-H1 regulation in the setting of the tumor microenvironment. Our results implicate that amphotericin B treatment interfered with the interactions between macrophages and Raji cells. Macrophages treated with amphotericin B reduced IL-4, IL-6, IL-10 and IL-13 synthesis in Raji cells, which were upregulated by M2 polarization. These cytokines are not only key cytokines that induce M2 polarization, but also the main factors for the upregulation of B7-H1 expression in the indirect co-culture system, especially IL-4 and IL-13. Several studies have investigated cytokines involved in B7-H1 regulation. Quandt et al. [28] reported that IL-4 could induce B7-H1 expression in renal cell carcinoma by activating the STAT6 signaling pathway. A direct binding site for STAT6 (TTACAAGAA) was found in the B7-H1 promoter by the promoter binding prediction program (TESS). In addition, IL-6 and IL-10 were reported to induce B7-H1 expression in circulating monocytes, TAMs and DCs, which were directly related to STAT3 activation [29, 30]. We assessed p-STAT3 and p-STAT6 in macrophages in the setting of the tumor microenvironment treated with or without amphotericin B. In our results, p-STAT3 and B7-H1 protein expression levels were not consistent; implying that the constitutive expression of B7-H1 in macrophages is independent of the STAT3 signaling pathway. B7-H1 and p-STAT6 protein expression in macrophages increased with time and the concentration of IL-4 and IL-13. We speculate that amphotericin B may regulate the IL-4/IL-13-STAT6 signaling pathway to inhibit the expression of B7-H1 protein in macrophages (Fig. 7). Whether STAT6 directly binds to the B7-H1 promoter warrants further studies.

Motoyoshi [25] reported a significant increase of iNOS protein and TNF-α mRNA in rat primary microglia treated with amphotericin B. In the present study, we observed that amphotericin B stimulated macrophages to synthesize IL-12 and TNF-α mRNA, which were mainly released by M1 macrophages; and amphotericin B simultaneously enhanced the phagocytic function of macrophages. Nevertheless, a change in M1 phenotype iNOS was not found. Taken together, our findings suggest that amphotericin B disrupts the tumor microenvironment by suppressing M2 phenotypes and B7-H1 rather than M1 polarization.

## Conclusions

In summary, in the co-culture system, Raji cells and macrophages co-operate to establish a pro-tumorigenic microenvironment. Amphotericin B has a potential value as an immune enhancer in BL treatment. The mechanism that amphotericin B disrupts the balance of the tumor microenvironment may be involved in the IL-4/IL-13-STAT6 B7-H1 signaling pathway.

## Additional files

Additional file 1 : **Figure S1.** The phenotype of immature macrophages is shown. CD14+ monocytes were isolated from PBMCs using CD14-microbeads and cultured with 5 ng/ml of GM-CSF for five days. These cells were analyzed for CD14, CD11b, CD11c, CD1a, CD83 and CD68 by flow cytometry. (TIFF 2137 kb)

Additional file 2 : **Figure S2.** Low doses (1 μM) amphotericin B is not toxic to macrophages. Macrophages were treated with increasing concentrations of amphotericin B for 24, 48 and 72 h, and cell viability was assessed by CCK-8 assay. (TIFF 62 kb)

## Abbreviations

BL: Burkitt’s lymphoma
DCs: Dendritic cells
IHC: Immunohistochemistry
NHL: Non-Hodgkin’s lymphoma
PD-L1: Programmed death ligand-1
TAMs: Tumor-associated macrophages
