# Supplementary figures and images for: Amphotericin B suppresses M2 phenotypes and B7-H1 expression in macrophages to prevent Raji cell proliferation
Source: BMC Cancer. 2018 Apr 26;18:467. doi: 10.1186/s12885-018-4266-0 (PMC5918564; doi:10.1186/s12885-018-4266-0)

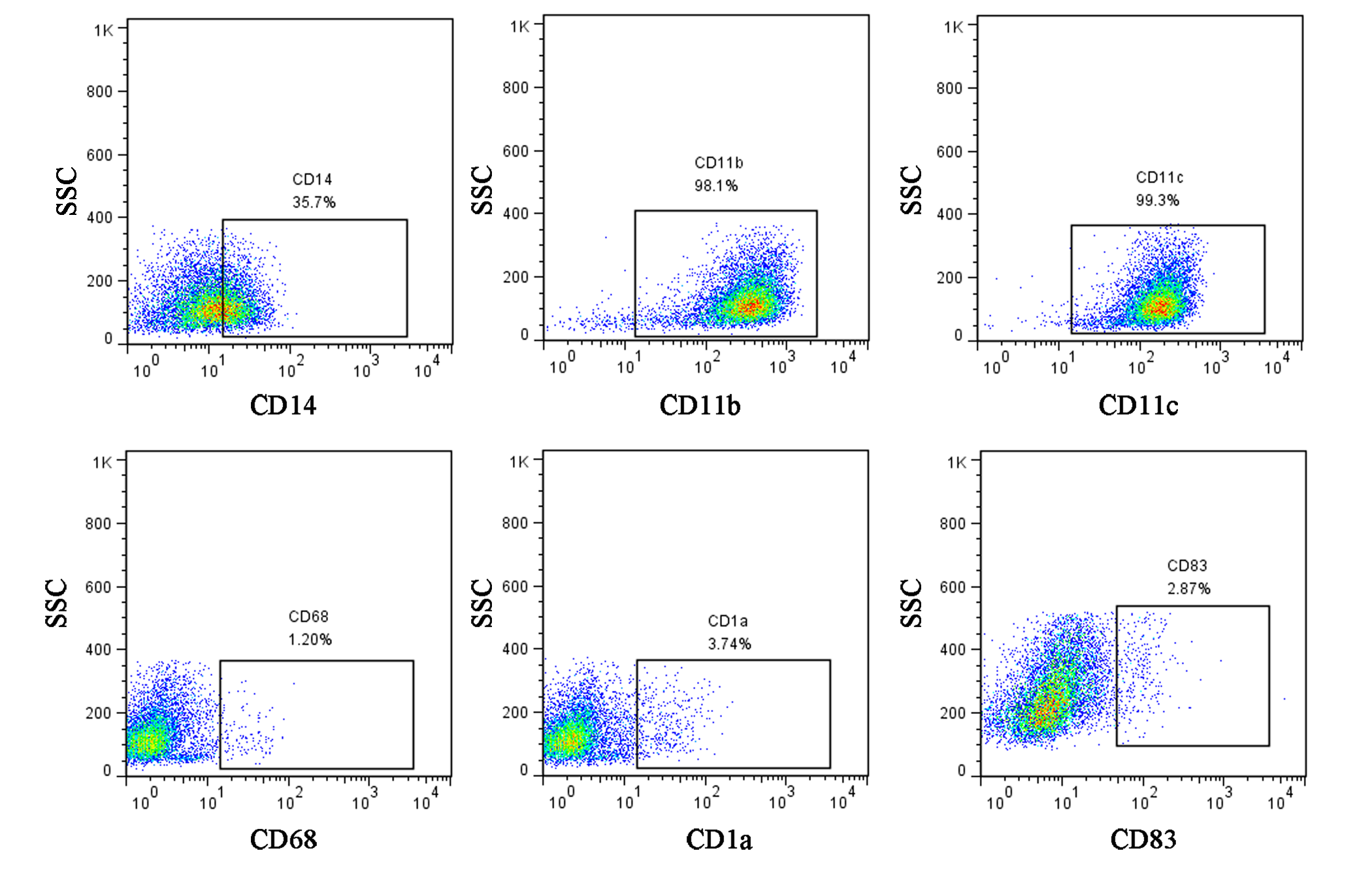

Supplement: Supplementary file 1 — : Figure S1. The phenotype of immature macrophages is shown. CD14+ monocytes were isolated from PBMCs using CD14-microbeads and cultured with 5 ng/ml of GM-CSF for five days. These cells were analyzed for CD14, CD11b, CD11c, CD1a, CD83 and CD68 by flow cytometry. (TIFF 2137 kb) [file 12885_2018_4266_MOESM1_ESM.tif]
